# Interaction between the intestinal flora and the severity of diversion colitis after low anterior resection of rectal cancer

**DOI:** 10.3389/fonc.2023.1001819

**Published:** 2023-03-14

**Authors:** Qiang Sun, Yunjie Shi, Xiaoben Liang, Hao Lu, Yu Huang, Lin Zhu, Wenqiang Wang, Wei Zhang, Zhiqian Hu, Xinxing Li

**Affiliations:** ^1^ Department of Anorectal Surgery, Changzheng Hospital, Naval Medical University (Second Military Medical University), Shanghai, China; ^2^ Department of General Surgery, Tongji Hospital, School of Medicine, Tongji University, Shanghai, China; ^3^ Department of Otolaryngology Head and Neck Surgery, Shanghai Children’s Hospital, School of Medicine, Shanghai Jiao Tong University, Shanghai, China; ^4^ Department of General Surgery, No.903 Hospital of PLA Joint Logistic Support Forcel, Hangzhou, China; ^5^ Department of Internal Medicine, Changzheng Hospital, Naval Medical University (Second Military Medical University), Shanghai, China

**Keywords:** oncology, colorecta cancer, surgery, colitis, intestinal flora

## Abstract

**Background:**

Diversion colitis (DC) is nonspecific inflammation of the distal intestinal mucosa following disruption of colonic continuity with colonic dysfunction. The colonscopic score is a good tool for differentiating the severity of patients with DC. At present, no studies have analyzed the pathogenesis of DC from the perspective of the diversity and and differences of intestinal flora.

**Methods:**

Retrospective study: Clinical information were collected from patients with low rectal cancer admitted to the Department of Anorectal Surgery, Changzheng Hospital, from April 2017 to April 2019. These patients underwent laparoscopic low anterior resection (LAR) combined with terminal ileum enterostomy (dual-chamber). We used chi-square test to comparethe clinical baseline information, clinical symptoms, and colonscopic characteristics between different severity of DC. Propsective oberservational study: We recruited 40 patients with laparoscopic anterior low resection combined with terminal ileum enterostomy and they were further classified into mild group and severe group according to the scores of colonscopic examinations for DC. 16s-rDNA sequencing was carried out to analyze the diversity and and differences of intestinal flora in the intestinal lavage fluid of the two groups.

**Results:**

In retrospective study, we found that age, BMI, history of diabetes, and symptoms associated with the stoma state were the independent risk factors that affect DC severity (*P*<0.05). Meanwhile, age, BMI, history of diabetes and colonscopic score were found to be independent risk factors affecting the severity of diarrhea after ileostomy closure surgery(*P<*0.05), which was consistent with our results of differentiating the severity of DC under endoscopy; In propsective oberservational study, 40 patients with low rectal cancer recruited by sample size calculation, 23 were in the mild group and 17 in the severe group. The results of 16s-rDNA sequencing showed that intestinal flora with high enrichment values primarily consisted of *Bifidobacteriales* and *Prevotella* in mild group, whereas that in the severe group consisted of *Providencia* and *Dorea*. The functional predictions on such two types of intestinal flora were mainly focused on lipid synthesis, glycan synthesis, metabolism, and amino acid metabolism pathways.

**Conclusion:**

After ileostomy closure surgery, a series of severe clinical symptoms might appear in DC patients. There are significant differences in local and systemic inflammatory responses, composition of intestinal flora between DC patients with different colonscopic scores, which provide a basis for the clinical interventional treatment for DC in patients with permanent stoma.

## 1 Introduction

According to 2020 cancer burden data, colorectal cancer is the third most common cancer and the second leading cause of cancer death worldwide, of which rectal cancer accounts for one-third (1). Radical excision remains the major clinical treatment for rectal cancer at present, and low anterior resection (LAR) has been increasingly widely applied in patients with low rectal carcinoma (2). Protective ileostomy is always applied to avoid clinically significant anastomotic leakage and other postoperative complications for patients receiving LAR surgery (3). Although ileostomy plays a protective role in anastomotic stoma, it may artificially lead to an abnormal diversion of digestive tract contents and cause diversion colitis (DC). In specific, DC is a newly proposed non-specific inflammation in the intestinal mucosa with colonic dysfunction (4). DC presents with erythema, diffuse granularity and indistinct vascular patterns under electronic colonoscop (5). It is also associated, to varying degrees, with mucosal fragility (80%), edema (60%), aphthous ulcers, and bleeding. With the time prolongation of stoma state, the condition of DC may become increasingly severe. The pathological features of DC include lymphoid follicular hyperplasia, intestinal mucosal atrophy, muscularis mucosal hypertrophy, Paneth cell metaplasia, diffuse active mucosal inflammation with crypt abscess (6). The pathogenesis of DC in patients with enterostomy status is unclear yet, although it may be related to intestinal bacteria disorder, insufficient short-chain fatty acids, and immune-inflammatory factors. In our clinical work, we found that the colonoscopic manifestations under the stoma were consistent with the diarrhea after ileostomy closure surgery. We were committed to verifying the accuracy of colonoscopic scores and explaining the differences in the severity of DC from the perspective of intestinal flora.

In this study, the clinical characteristics of patients with enterostomy status-related DC were observed and analyzed. We explored the correlation between intestinal flora imbalance and DC developmentand and elucidated the possible mechanisms. This study consists of two major parts: (1) Retrospectivly analyzed the clinical characteristics of patients with DC and verified the accuracy of colonoscopic scores; (2) Prospectively analyzed the relationship between intestinal flora and DC after ileum enterostomy. We are looking forward to the clinical interventional treatment of enterostomy-related DC from the perspective of regulating intestinal flora, with the focus on alleviating the clinical symptoms of such patients.

## 2 Retrospective study

### 2.1 Sample sources and methods

This study enrolled 305 patients with rectal cancer admitted in our center from April 2017 to April 2019. They all underwent laparoscopic LAR for rectal cancer. Among them, 167 patients were combined with concurrent terminal ileum enterostomy (dual-chamber) to prevent anastomotic leakage. Ethical review number was as follows: ChiECRCT-20180225.

Inclusion criteria: ①Diagnosed with rectal cancer through enteroscopy and pathology; ② Clinical stage III or below; ③ Complete medical data and receiving follow-up visits for at least 24 weeks.

Exclusion criteria: ① Combined with other infectious enteritis, autoimmune enteritis, radiation enteritis, or inflammatory bowel diseases; ② Follow-up was inconclusive or nonsurvivable; ③ Taking hormones, antibiotics, or immunosuppressors ≥1month in the course of the disease; ④ Postoperative C grade anastomotic leakage or infectious peritoneal effusion; ⑤ Other gastrointestinal tumors or surgical history; ⑥ Neoadjuvant or postoperative radiotherapy; ⑦ Vegetarians.

In this study, we performed a three-step retrospective comparative analysis of the risk factors of different severity of DC and diarrhea after ileostomy closure surgery. Step 1: According to colonscopic score (**Table S1**), 110 patients met the requirements of the study were divided into the mild group (52 cases) and the severe group (58 cases). The follow-up time was 3-6 months. The general data differences between the two groups were compared and analyzed. Step 2: Among 110 patients, 85 patients underwent ileostomy closure surgery. 25 patients were excluded because they could not be operated again due to age, underlying diseases and other reasons and became permanent stoma condition. 45 patients remained in the mild group and 40 patients remained in the severe group. The general data differences between the two groups after ileostomy closure surgery were compared and analyzed. Step 3: After ileostomy closure surgery, the most prominent clinical symptom is diarrhea. Then we wanted to examine differences in general data for patients with different degrees of diarrhea. We used the 20-point scoring method to distinguish the severity of diarrhea in 85 patients after ileostomy closure surgery within 6 month, and finally divided them into 41 cases of mild diarrhea group and 44 cases of severe diarrhea group. The differences of general data between the two groups were compared and analyzed.

### 2.2 Relevant diagnostic criteria

Colonscopic scores are detailed as follows. Colonoscopic findings include ulcer, follicular hyperplasia, anastomotic stenosis, inflammatory polyp, and mucosa edema (7). The colonoscopic rating (8, 9) consists of three elements of edema (0–3 points), mucosal hemorrhage (0–3 points), and contact hemorrhage (0–1 point). The total score was obtained by adding the points described above. On the basis of the total score, mild (0–3 points) or severe (4–7 points) symptoms were distinguished (**Table S1** and **Figure S1**). On the basis of their colonscopic scores, the patients with DC were divided into a group of mild symptoms (the mild group) and a group of severe symptoms (the severe group).

For all patients, their clinical symptoms (e.g., abdominal pain, mucoid stool, and haematochezia) subsequent to LAR as well as those (e.g., tenesmus, abdominal pain, anal pain, diarrhea frequency, time for defecation return to normal, mucous stools and haematochezia) within 6 months after ileostomy closure surgeries were collected and conducted statistical analysis.

Diarrhea severity rating is described as follows. After ileostomy closure surgery, the most prominent clinical symptom is diarrhea (10). This study selected 20-point scoring (11) to distinguish the severity of diarrhea after the ileostomy closure surgery, covering duration and frequency of diarrhea, vomiting duration, fever and dehydration state (estimated by a ratio of weight loss to the total weight). A higher score indicates more severe diarrhea, and more than 10 points suggests mild diarrhea, whereas that above 10 points indicates severe diarrhea (**Table S2**).

The severity rating system of the International Rectal Cancer Study Group classifies anastomotic leakage after rectal surgery into three grades: A, B and C. Crade A is relatively light, without special treatment; Grade B requires intervention but does not require open surgery; Grade C requirs reoperation, which is a relatively serious complication. Reoperation has a great impact on the intestinal environment. So the patients with grade C anastomotic leakage were excluded in this study.

## 3 Propsective oberservational study

### 3.1 Sample sources and methods

We found that colonscopic score was an independent risk factor for diarrhea severity after ileostomy closure surgery. Therefore, in prospective studies, we continued to use colonscopic score to distinguish the severity of DC patients. According to the requirements of the case-control study, patients meeting the requirements of the study were prospectively selected in our center from August 2019 to August 2020. All patients underwent laparoscopic LAR combined with terminal ileum enterostomy and returned to our hospital 3 months after surgery. Colonoscopies were performed by the same investigator to assess the severity of DC. Mechanical bowel preparation was not performed before colonoscopy in order to reduce bias. The Olympus colonoscopy system was used to conduct full-layer examination of putting-aside colon and take images through anus. In this study, all patients presented with varying degrees of DC, and the colonscopic scoring requirements and methods were the same as before. The general data differences between the two groups were compared and analyzed. This study has been approved by the Ethics Committee of Changzheng Hospital, the Ethical review number was as follows:ChiECRCT-20190233, and the informed consent of patients was obtained.

Based on relevant literature and clinical practice, this study intended to include about 8 variables for data analysis. In multi-factor non-conditional logistic regression analysis, the sample size of dependent variables with a low incidence was required to be at least 5-10 times that of the included variables(5 times in this study). In addition, according to previous research results, the incidence of DC was 100%, so the total sample size of this study was 8×5÷100%=40 cases.

### 3.2 Calprotectin test in anal lavage fluid

Anal lavage fluid samples were selected and sampled prior to endoscopy to ensure that the results were not affected by endoscopy. Six hours before colonoscopy, 250ml normal saline was retained from the anus by infusion strip for enema, and 50-100ml of enema was retained and stored in the refrigerator at -80°C. The content of calprotectin in the anal lavage fluid of the two groups was standardized according to the requirements of the kit instruction (Shanghai Zeye Biotechnology Co., LTD.).

### 3.3 Inflammatory factor level test

Under colonoscopy, biopsy forceps were used to take 3-4 pieces of mucosal tissue with the most severe inflammation as tissue samples. 10ml of fasting venous blood was extracted from the patients in the morning, heparin was treated with anticoagulation, and centrifuged at 5000r/min at 4°C for 5min. The serum was separated and frozen at -80°C for testing. After collection, sterile diluent was added for 10-fold dilution, and the tested solution was cultured in selective lactobacillus culture medium (LBS) at 37°C and anaerobic conditions for 72h. After the culture, the contents of TNF-α, IL-1β, IL-6 and IL-17 in tissues and plasma were detected by enzyma-linked immunosorbent assay (ELISA), and the operation was standardized according to the kit instruction (Shanghai Qiyi Biotechnology Co., LTD.).

### 3.4 Lipopolysaccharide test

The plasma sampling method was the same as above. The content of LPS in plasma was detected by ELISA, and the standard operation was carried out according to the kit instruction (Shanghai Xinyu Biotechnology Co., LTD.).

### 3.5 16S rRNA sequencing and analysis for anal lavage fluid

Anal lavage fluid method see above. Samples are transported to the laboratory as soon as possible and all operations are carried out in the anaerobic chamber (atmospheric environment:Nitrogen (75%), hydrogen (10%) and carbon dioxide (15%) were inoculated on two blood AGAR plates (BAP) for 24h at 35°C under anaerobic conditions in a 5% CO2 incubator. Brucella culture medium was incubated in an anaerobic tank for 48h, and the species of cultured bacteria were identified qualitatively. Based on BGI, 16S rRNA sequencing of anal lavage fluid was performed. The main steps were as follows:DNA extraction, PCR amplification and library construction. HiSeq platform was selected for sequencing of qualified libraries according to the inserted fragment size.

Next, bioinformatics analysis, such as extraction level analysis, Operational Taxonomic Unit (OTU) clustering analysis, species diversity analysis, species composition analysis and species difference analysis, was carried out. Parallel function prediction:PICRUSt software was used to standardize OTU abundance table first, that is, to remove the influence of copy number of 16S marker gene in species genome. Then, KO corresponding to OTU was obtained by greengene ID corresponding to each OTU, and the abundance of KO was calculated by the sum of OTU abundance corresponding to KO.

## 4 Statistical methods

SPSS (26.0, IBM) statistical software was selected to complete data entry and statistical analysis. General data of patients in both groups were descriptively analyzed. Quantitative data were denoted by ( $\bar{x}\pm s$ ), and inter-group comparison was conducted by t test for 2 independent samples. Relevant enumeration data were expressed in percentage (%), and χ^2^ test was carried out for comparison between the two groups. The severity of DC and general clinical data were analyzed by univariate analysis. The significant factors in univariate analysis were used as independent variables, and the severe DC was used as dependent variable for Logistic multiple analysis by stepwise method. Odds ratio (OR) was used to represent the strength of association between the two factors (OR > 1 was a risk factor, OR< 1 was a protective factor). The results of intestinal flora in both groups were subjected to Wilcoxon rank-sum tests on the HiSeq platform. Statistical significance was considered at *P*<0.05.

## 5 Results

### 5.1 The result of retrospective study

In step 1, there were statistically significant differences in age, BMI, diabetes and incidence of DC-related clinical symptoms (including abdominal pain, mucous stools and haematochezia) between two groups (*P*<0.05). The incidence of abnormal colonoscopic manifestations (including ulcers, follicular hyperplasia, inflammatory polyps, mucosal edema, anastomotic stenosis, etc.) in the severe group was significantly higher than that in the mild group (*P*<0.05), which was consistent with the results of colonscopic score grouping. Age, BMI, diabetes, symptoms of stoma state were independent risk factors influence the severity of the DC (all *P*<0.05) (**Table 1**).

**Table 1:** Univariate and multiple analysis of clinical data for two groups in stoma status (n/%).

Parameter	Characteristic	Mild group (n=52)	Severe group (n=58)	Univariate analysis		Multiple analysis	
				Log rank χ^2^ test	P	OR (95%CI)	P
Gender				2.314	0.468	–	NI
	Male	32 (61.54)	39 (67.24)				
	Female	20 (38.46)	19 (32.76)				
Age (years)				9.454	0.009		<0.0001
	≤60	35 (67.31)	28 (48.28)			Reference	
	>60	17 (32.69)	30 (51.72)			0.708 (0.658~0.762)	
BMI (kg/m^2^)				5.356	0.019		<0.0001
	<25	33 (63.46)	30 (51.72)			Reference	
	≥25	19 (36.54)	28 (48.28)			0.771 (0.644~0.923)	
Diabetes		5 (23.63)	20 (34.48)	13.234	0.004	0.895 (0.708~1.132)	<0.0001
Pathological stage				1.564	0.472	–	NI
	I~II	34 (65.38)	39 (67.24)				
	III	18 (34.62)	19 (32.76)				
Postoperative chemotherapy				1.378	0.482	–	NI
	Yes	16 (30.77)	16 (27.57)				
	None	36 (69.23)	42 (72.41)				
Symptom of stoma state				8.420	0.010		<0.0001
	Abdominal pain	6 (11.54)	18 (31.03)			Reference	
	Mucoid stool	3 (5.77)	12 (20.69)			1.029 (0.800~1.323)	
	Haematochezia	1 (1.92)	6 (10.34)			1.288 (1.041~1.594)	
Colonoscopic findings				37.379	<0.0001		<0.0001
	Ulcer	5 (9.62)	17 (29.31)			Reference	
	Follicular hyperplasia	10 (19.23)	29 (50.00)			0.864 (0.787–0.948)	
	Inflammatory polyp	10 (19.23)	28 (48.28)			0.942 (0.832–1.065)	
	Mucosal edema	42 (80.77)	58 (100.00)			1.087 (0.928–1.208)	
	Anastomotic stenosis	1 (1.02)	6 (15.52)			1.180 (0.946–1.301)	

NI, not included in the multiple analysis.

In step 2, the incidence of postoperative clinical symptoms (including abdominal pain, haematochezia, tenesmus, anal pain, etc.) in severe group after ileostomy closure surgery was significantly higher than those in mild group (*P*<0.05). CRP, diarrhea frequency per day and time for defecation return to normal in severe group were significantly higher than those in mild group (*P*<0.05) (**Table 2**).

**Table 2:** Analysis of Clinical follow-up data of two groups after closure of temporary ileostomy (n/%, $\bar{x}\pm s$ ).

Indicators	Characteristic	Mild group (n=45)	Severe group (n=40)	P
Clinical signs and symptoms				
	Abdominal pain	25 (55.56)	35 (87.50)	0.001
	Haematochezia	10 (22.22)	18 (45.00)	0.022
	Tenesmus	8 (17.78)	20 (50.00)	0.013
	Anal pain	4 (8.89)	13 (32.50)	0.005
Diarrhea frequency per day		4.14±1.69	11.21±4.27	0.007
Time for defecation return to normal (d)		15.67±4.78	30.92±9.09	0.003
Complete blood count				
	WBC (×10^9^/L)	6.44±3.42	7.08±1.34	0.188
	NEUT% (%)	68.43±6.32	70.4±5.13	0.101
	CRP (mg/L)	8.82±1.96	20.07±5.99	0.004

In step 3, the age and BMI of patients with severe diarrhea group were significantly higher than those with mild diarrhea group, and the incidence of diabetes, abdominal pain, mucus stool, haematochezia and other abnormal symptoms in patients with severe diarrhea were significantly higher than those with mild diarrhea (*P*<0.05). Age, BMI, diabetes and colonscopic score were independent risk factors for diarrhea severity (*P*<0.05) (**Table 3**).

**Table 3:** Analysis of clinical data of patients with different diarrhea degree (n/%).

Parameter	Characteristic	Mild diarrhea group (n=41)	Severe diarrhea group (n=44)	Univariate analysis		Multiple analysis	
				Log rank χ^2^ test	P	OR (95%CI)	P
Gender				1.678	0.577	–	NI
	Male	26 (63.41)	28 (63.64)				
	Female	15 (36.59)	16 (36.36)				
Age (years)				5.321	0.020		<0.0001
	≤60	16 (39.02)	8 (18.18)			Reference	
	>60	25 (60.98)	36 (81.82)			0.802 (0.622–1.034)	
BMI (kg/m^2^)				35.435	<0.0001		<0.0001
	<25	25 (60.98)	17 (38.64)			Reference	
	≥25	16 (39.02)	27 (61.36)			1.224 (0.930–1.611)	
Diabetes		6 (14.63)	18 (40.91)	8.224	0.005	0.890 (0.626–1.263)	0.005
Symptom of stoma state				4.345	0.054	–	NI
	Abdominal pain	9 (21.95)	14 (31.82)				
	Mucoid stool	6 (14.63)	10 (22.73)				
	Haematochezia	5 (12.20)	8 (18.18)				
Postoperative chemotherapy				3.679	0.086	–	NI
	Yes	17 (41.46)	13 (29.55)				
	None	24 (58.54)	31 (70.45)				
colonscopic score				73.80	<0.0001		<0.0001
	Mild	35 (85.37)	10 (22.73)			Reference	
	Severe	6 (14.63)	34 (77.27)			0.996 (0.840–1.182)	

NI, not included in the multiple analysis.

### 5.2 The result of propsective oberservational study

#### 5.2.1 Comparison of general data, calprotectin, inflammatory factors, and LPS between the two groups

**Figure 1:**
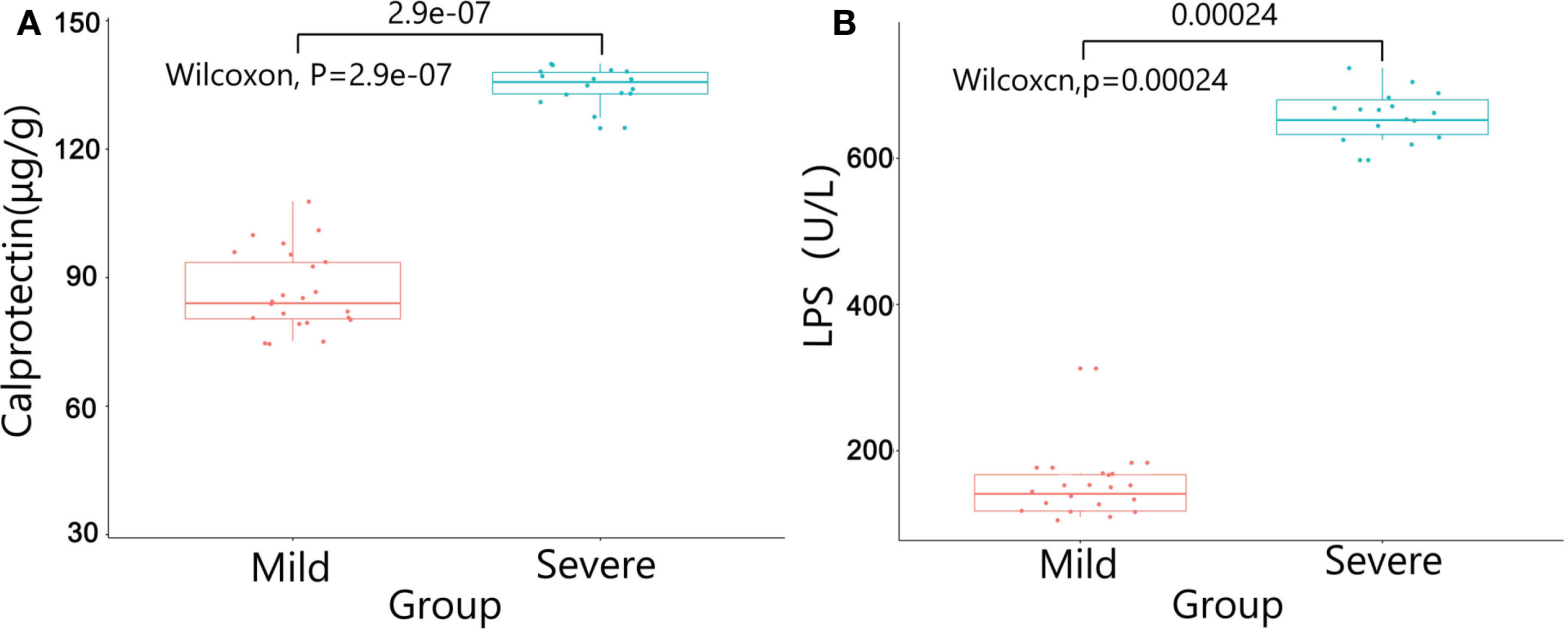
The results of Calprotectin and LPS between the two groups. **(A)** Calprotectin in anal lavage fluid; **(B)** LPS in plasma.

**Figure 2:**
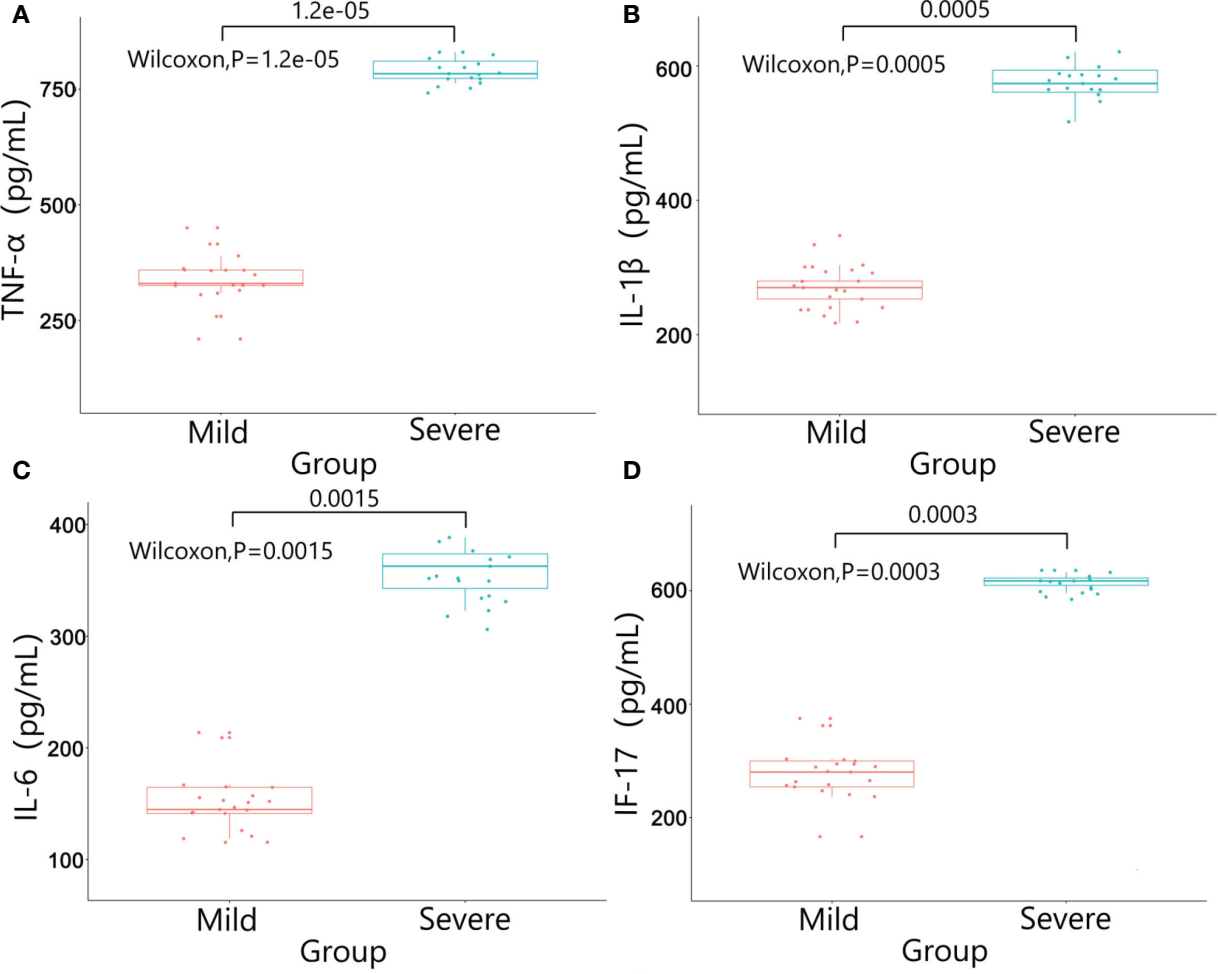
Results of inflammatory factors in colon wall tissue between the two groups. **(A)** TNF-α; **(B)** IL-1β; **(C)** IL-6; **(D)** IL-17.

**Figure 3:**
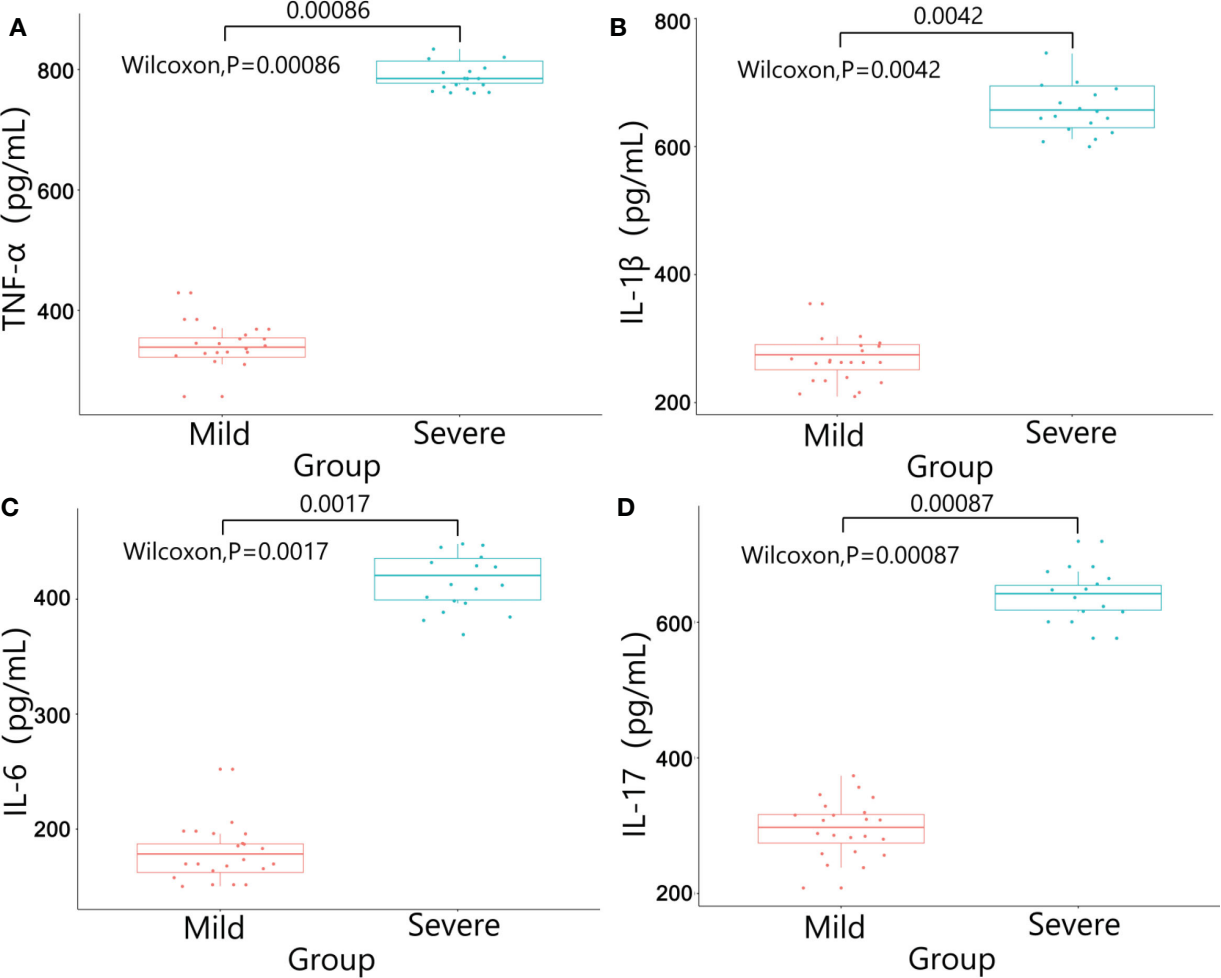
Results of inflammatory factors in plasma between the two groups. **(A)** TNF-α; **(B)** IL-1β; **(C)** IL-6; **(D)** IL-17.

There were no significant differences in baseline data such as gender, age, BMI, diabetes, pathological stage, chemotherapy regimens and duration of ostomy between two groups (*P*>0.05). The patients were followed up after ileostomy closure surgery, and the severity of diarrhea in the two groups and the CRP results one month after ileostomy closure surgery were significantly different (*P*<0.05)(**Table 4**). The content of calprotectin in anal lavage fluid in the severe group was significantly higher than that in the mild group, with significant difference between the two groups (*P*<0.05)(**Figure 1A**), as shown in **Figure 1B**. The plasma LPS level of the severe group was significantly higher than that of the mild group, with significant difference between the two groups (*P*<0.05)(**Figure 1B**). The results showed that the levels of TNF-α, IL-1β, IL-6 and IL-17 in the severe group were significantly higher than those in the mild group (*P*<0.05)(**Figure 2**). The results showed that the levels of TNF-α, IL-1β, IL-6 and IL-17 in the severe group were significantly higher than those in the mild group (*P*<0.05) (**Figure 3**).

**Table 4:** Comparison of baseline data between two groups (n/%, $\bar{x}\pm s$ ).

Indicators	Characteristic	Mild group (n=23)	Severe group (n=17)	P
Gender				0.226
	Male	13 (56.52)	10 (58.82)	
	Female	10 (43.48)	7 (41.18)	
Age (years)				0.194
	≤60	9 (39.13)	7 (41.18)	
	>60	14 (60.87)	10 (58.82)	
BMI (kg/m^2^)				0.082
	<25	13 (56.52)	10 (58.82)	
	>≥25	10 (43.48)	7 (41.18)	
Diabetes		3 (13.04)	2 (11.76)	0.132
Pathological stage				0.157
	I-II	14 (60.87)	11 (64.71)	
	III	9 (39.13)	6 (35.39)	
Chemotherapy regimens				0.091
	Xelox	3 (13.04)	2 (11.76)	
	Xeloda only	1 (4.35)	1 (5.88)	
	FOLFIRI	4 (17.39)	3 (17.65)	
	None	15 (65.22)	11 (64.71)	
Duration of ostomy state (month)		5.87±1.44	6.08±1.90	0.098
The severity of diarrhea(After stoma reversal)				
	Mild diarrhea	20 (86.98)	1 (5.88)	<0.0001
	Severe diarrhea	3 (13.04)	16 (94.12)	
CRP (mg/L)(After stoma reversal)		2.22±0.51	11.21±2.91	0.020

#### 5.2.2 OTU clustering

**Figure 4:**
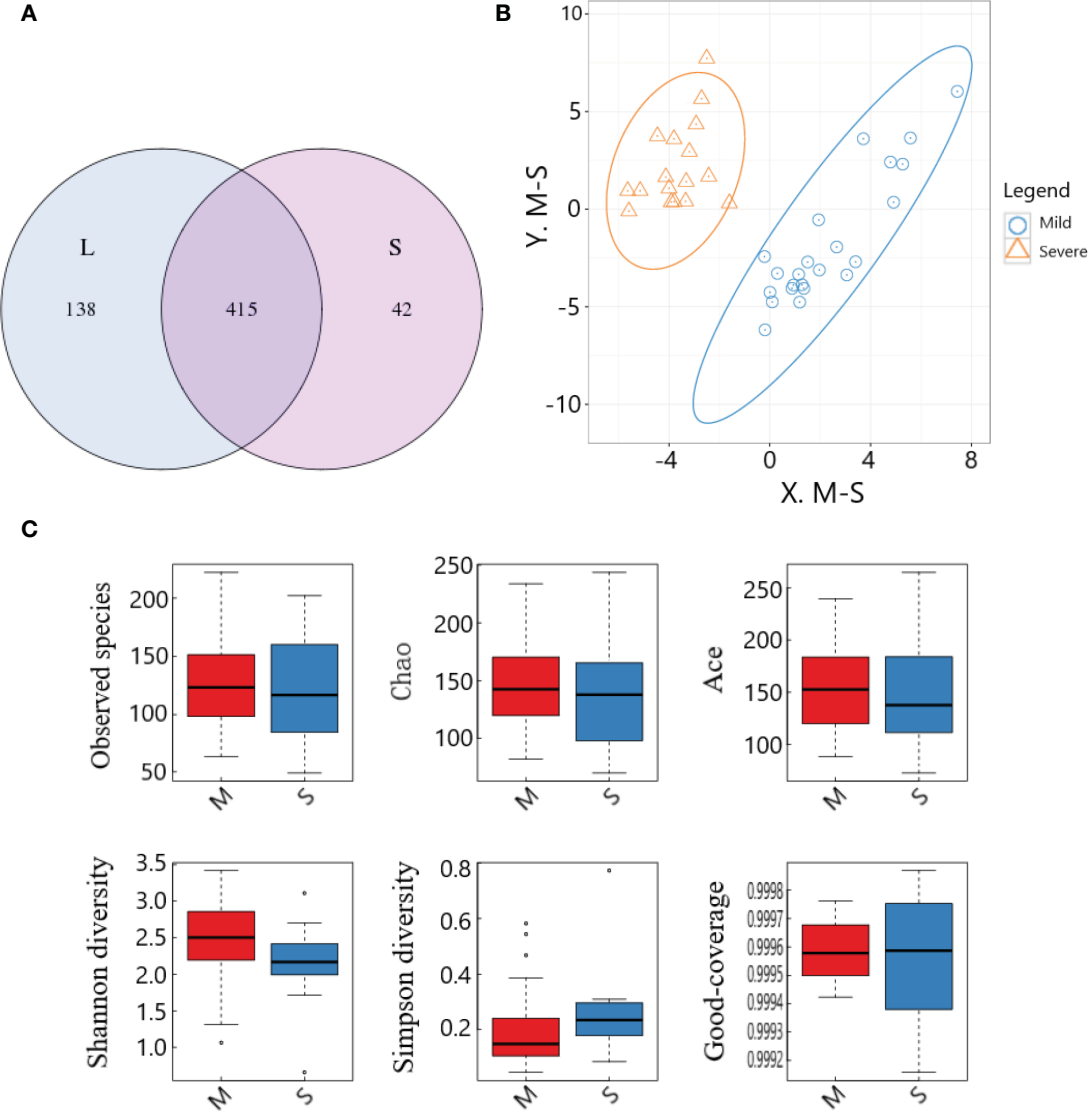
OTU cluster analysis and species diversity analysis of samples between the two groups. **(A)** OTU Venn diagram; **(B)** PLS-DA analysis; **(C)** Comparison of α-diversity.

The lowest sequence number in all samples was extracted to level, and the OTU number statistics of each sample were divided into 97% sequence similarity level(**Table S3**). The sequence of original data after splicing and quality control was analyzed to figer out the overlapping of OTU clustering. The results showed that a total of 595 different OTUs were obtained, among which 415 otUs were shared by the two groups (**Figure 4A**).

#### 5.2.3 Species diversity analysis for intestinal flora

PLS-DA analysis was performed on the OTU results of the two groups of samples, and the results showed that the distribution characteristics of the two groups were relatively concentrated within the group and relatively dispersed between the groups. There was no obvious overlap between the two groups, and there was not much overlap between the two groups, and the overall distribution was slightly dispersed. The two groups had good aggregation and differentiation respectively, indicating that there were significant differences in the bacterial community structure of the two groups (**Figure 4B**).

In this study, box plots were made for the six indices of the two groups of specimens respectively, which could intuitively evaluate the data distribution characteristics. The results showed that, on the whole, most of the median were distributed near the center of the box, indicating that the data distribution was relatively symmetrical, and the mean could be calculated for quantitative comparison. Observed species, Chao, Ace and Shannon indexes in the severe group were significantly lower than those in the mild group, while Simpson index was significantly higher than that in the mild group (all *P*<0.05), suggesting that the intestinal microflora data in the severe group was more concentrated, but the diversity was significantly lower than that in the mild group. Good-coverage values of both groups were close to 1 (**Figure 4C**), suggesting that the results of this study were fairly representative of the actual situation.

#### 5.2.4 Species annotation analysis and composition differences of intestinal flora

**Figure 5:**
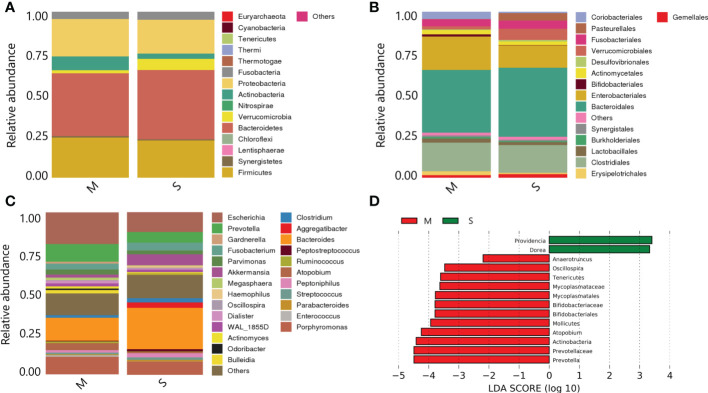
Species annotation analysis and composition differences of samples between the two groups. **(A)** On phylum level; **(B)** On order level; **(C)** On family level; **(D)** Histogram of LDA value distribution.

(1) Species composition at phylum level:*Bacteroidetes* and *Firmicutes* were the dominant phyla with the highest overall abundance. *Proteobacteria* and *Actinobacteria* followed, and the total relative abundance of these four phyla exceeded 90%. *Bacteroidetes* and *Firmicutes* were the main components of intestinal flora. The *Actinobacteria* in the severe group was significantly lower than that in the mild group (*P*<0.05), while the difference of *Bacteroidetes* and *Firmicutes* between the two groups was small (P>0.05) (**Figure 5A**).

(2) Species composition at order level:*Bacteroidales*, *Clostridiales* and *Enterobacteriales* were the most abundant in the samples. *Bifidobacteriales* in the severe group were significantly lower than those in the mild group (*P*<0.05) (**Figure 5B**).

(3) Species composition at genus level:*Bacteroides*, *Escherichia* and *Porphyromonas* were the dominant genera with the highest overall abundance. The *Prevotella* and *Escherichia* levels in the severe group were significantly lower than those in the mild group (*P*<0.05) (**Figure 5C**).

(4) LefSe analysis of species composition differences:The histogram showed the results of LefSe analysis visually (all species shown in the figure were LDA score≥2, with statistical differences). Among them, *Bifidobacteriales*, *Mollicutes*, *Atopobium*, *Prevotella* and *Actinobacteria* were the most abundant in the mild group. In the severe group, *Providencia* and *Dorea* had higher abundance (**Figure 5D**).

#### 5.2.5 Function prediction

**Figure 6:**
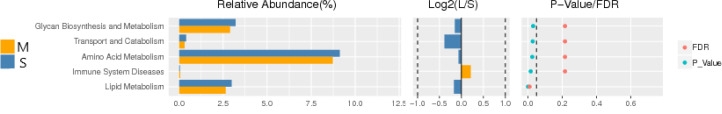
Diagram of the difference in the Keeg Path Wilcox Test between the two groups.

Function prediction of 16S-RDNA is to standardize OTU abundance table by PICRUSt, obtain KO information and abundance corresponding to OTU corresponding to Greengene ID, and predict genes for KEEG function classification prediction. There were significant differences between the two groups in genes enriched in glycan synthesis and metabolism, amino acid metabolism, immune system diseases, lipid synthesis and other metabolic pathways (*P*<0.05) (**Figure 6**).

## 6 Discussion

For patients with low rectal cancer (distance from inferior margin of the tumor to the anal edge≤5 cm), concurrent prophylactic terminal ileostomy is often considered during LAR surgery for rectal cancer. Terminal ileostomy could effectively protect rectal anastomosis from anastomotic leakage and reduce complications caused by poor healing of rectal anastomosis. However, studies in recent years has shown that DC is common in patients after terminal ileostomy. DC is also clinically known as disuse colitis (12). DC was first mentioned by Basil Morson et al. (13) in 1974, when it was described as a segmental nonspecific inflammation confined to the colonic mucosa. David Glotzer et al. (14) published a case study of 10 patients in 1981, and defined this inflammation as DC. However, the exact mechanism of this disease has not yet been clarified.

Because enterostomy related-DC patient is in a stoma state, the typical clinical symptoms of DC are generally not obvious. For this reason, the actual incidence rate of DC might be underestimated. According to studies (15), the most common symptoms in DC patients are serous, bloody, or mucous stools (40%), followed by abdominal pain and tenesmus (15%). In this study, the incidence rate of abdominal pain, mucus stool, and bloody stool were lower than other studies. The possible reason is that our study was a retrospective study, and there might be a certain bias in the patient’s impression of previous symptoms. Although enterostomy closure surgery is the best method for the treatment of DC, the short-term clinical symptoms after restoration are usually obvious, which needs our attention. In addition, there are still a considerable number of patients who receive stomostomy, due to age, physical conditions and other reasons, cannot receive ileostomy closure surgery, forming permanent stomostomy, and the related symptoms will affect the quality of life, which is also the purpose of our next study. Our study not only focused on symptoms after ileostomy closure surgeries (e.g., abdominal pain, severe diarrhea, bloody stools, tenesmus, and anal pain), but also attached great importance to operation-related infections, enterocutaneous fistula, anastomotic leakage, anastomotic stenosis, and interstitial abscess. Nevertheless, no commonly used disease diagnosis standards are available globally (16). In this study, the severity of DC was graded by colonscopic scores, and we found that age, BMI, history of diabetes, and enterostomy symptoms were independent risk factors for the severity of diarrhea, which provided data support for clinical prediction of the incidence of DC.

The pathogenesis of DC is not yet clear, which may be related to intestinal bacteria disorder, lack of SCFA and immune inflammatory factors (17). Some studies have found that the lesions of the colonic mucosa might be caused by metabolites (18). Metabolites in feces are an important source of energy for the body, and disruption of fecal flow may lead to colitis, but direct evidence is still few (19). Glotzer et al. (12) speculated in a clinical report about 10 DC patients that DC might be the result of factors such as excessive proliferation of pathogenic bacteria, nutritional deficiencies, toxins, or disturbance of the symbiotic relationship between bacteria and the mucosal layer in gut. In an *in vivo* study (20), it was shown that the changes in gut microbiota were dominated by the increase of *Bacteroides* and *Clostridium*. It was speculated that bacteria were important environmental factors in the pathogenesis of DC, and the changes of gut microbiota may be involved in the pathogenesis of DC. One study showed (21, 22) decreased concentrations of carbohydrate-fermenting anaerobic and pathogenic bacteria in dysfunctional colon, which might suggest that the overproliferation of anaerobic or pathogenic bacteria is not important causative factors. Additional studies have shown that nitrate-reducing bacteria are increased in the gut of DC patients. Nitric oxide (NO) produced by nitrate-reducing bacteria has a dual effect on colon tissue, with a protective effect when NO concentration is low, but a toxic effect when the concentration is high (23). Therefore, the increase of nitrate-reducing bacteria might increase the toxicity level of intestinal tissue, and then trigger DC (24). In addition, current epidemiological studies (25) have not found specific pathogenic bacteria that cause DC, and the clinical effect of antibiotic therapy is poor. Therefore, DC may not be caused by direct infection by pathogenic bacteria, suggesting that changes in gut microbiota may only be part of environmental factors (26).

In this study, we used colonscopic scores to grade the severity of DC patients, and calprotectin, inflammatory factors, plasma lipopolysaccharide were tested to verify the severity of DC. Calprotectin was one kinds of calcium-binding protein whose expression levels were often abnormally increased during inflammatory responses. Therefore, calprotectin could be used as a marker of inflammatory cell activation and might be abnormally increased in a variety of enteritis (27). In this study, the expression level of calprotectin in the intestinal enema fluid of the severe group was significantly higher than that of the mild group (P<0.05), suggesting that calprotectin could be used as a detection method to evaluate whether DC was in the active phase. The chemical nature of LPS was endotoxin, the important component of the cell wall of Gram-negative (G^-^)bacteria. In the condition of severe infection, endogenous LPS in the intestine entered the blood through the intestinal wall, and excessive intestinal LPS might cause abnormal intestinal metabolism and immune response (28). In our study, the plasma LPS level in the severe group was significantly higher than that in the mild group, suggesting that the intestinal wall permeability was increased and the intestinal barrier was damaged in patients with severe DC. The onset and progression of DC might be closely related to the inflammatory response. The abnormal expression of inflammatory factors such as TNF-α, IL-1β, IL-6 and IL-17 might affect the pathophysiological processes such as intestinal flora imbalance and immune dysfunction in DC patients (29). In addition, inflammatory factors could invade the mucosal layer, damage the intestinal mucosal barrier, aggravate the damage of intestinal epithelial tissue, and lead to the recurrence of DC disease (30). In this study, the expressions of inflammatory factors in the intestinal mucosal tissue and plasma of the severe group were significantly higher than those of the mild group. The possible reason was that the excessive immune response aggravated the intestinal mucosal damage in DC patients, which lead to the increase of the permeability of the intestinal mucosa and the decrease of the barrier function.

Our study was the first to evaluate the relationship between gut microbiota and DC using bacterial culture combined with 16S rDNA sequencing. Moreover, In this study, the traditional sampling method was improved, and the method of intestinal enema was selected, which had a lower chance of being exposed to oxygen during the operation, and the temperature and humidity environment was controllable, and had better accuracy. We found that comparing the two groups of flora at the phylum level, the *Actinomycete phylum* in the severe group was significantly lower than that in the mild group. However, there was no difference in *Firmicutes* and *Bacteroidetes* between the two groups. In the comparison of the two groups of bacteria at the order level, *Bifidobacteriales* in the severe group were significantly lower than that in the mild group. The comparison of the two groups of flora at the *genus* level showed that *Prevotella* and *Escherichia* in the severe group were significantly lower than that in the mild group. The results of LefSe analysis showed that the bacteria with higher abundance in the mild group were mainly *Bifidobacteriales*, *Mollicutes*, *Atopobium*, and *Prevotella*, *Actinobacteria*. The more abundant flora in the severe group were *Providencia* and *Dorea*. In addition, we compared and analyzed the 16S rDNA sequencing data of the intestinal flora of DC patients in two groups. The results showed that there were significant differences between the two groups in genes enriched in metabolic pathways such as glycan synthesis and metabolism, amino acid metabolism, immune system diseases and lipid synthesis. Our results suggested that the main reason for the occurrence of DC might be the decrease of beneficial bacteria or the increase of pathogenic bacteria.

*Bifidobacterium*, as representative gram-positive(G^+^) beneficial bacteria in *Actinomyces*, has functions including up-regulating systemic immune response, stimulating cellular immunity and preventing adverse infections, and is of great significance for participating in intestinal immunity and maintaining intestinal health (31). *Bifidobacterium* on DC is not yet clear, the possible mechanisms lie in (32, 33):1) *Bifidobacterium* can regulate the balance of intestinal flora, and by changing the pH value and substance metabolism of the intestinal environment, produce beneficial effects nutrients and antibacterial substances; 2) *Bifidobacterium* is beneficial to block the production of intestinal pathogenic bacteria (including *Escherichia coli*, *Salmonella*, etc.); 3) *Bifidobacterium* can be closely combined with intestinal epithelial cells through the adhesion effect to generate an effective biofilm barrier, which can effectively inhibit the invasion and transfer of intestinal pathogens;4) *Bifidobacterium* can secrete anti-inflammatory cytokines to regulate the process of immune system and inflammatory response. *Bacteroidetes* are the main components of the intestinal flora, and their presence plays a key role in the maintenance of intestinal health and material metabolism (34). *Prevotella* is a common probiotic in the *phylum Bacteroidetes*, which is beneficial to regulate immune response and protect the structural integrity of intestinal mucosa. Russell TA (35) performed genomic analysis on the stool of DC patients, and the results showed that the abundance of *Prevotella* in the gut of patients with severe DC was reduced, similar to the results of our research. The possible reason is that *Prevotella* is an important component involved in the metabolism of various substances such as sugar and lipid, and lack of *Prevotella* affect the stability of substance metabolism. Dysregulation of *Prevotella* may lead to gastrointestinal motility disorders, and patients with DC may present with clinical symptoms such as abdominal pain, nausea, and vomiting. *Providence* can cause infection in multiple organs, mainly urinary tract and colon, which also can lead to an outbreak of nosocomial infections (36). *Dorella* can affect the function of intestinal epithelial cells and increase the permeability of the intestinal epithelium, which leads to the entry of endotoxins into the blood and triggers chronic inflammation (37). In addition, the study of Abegunde AT (38) showed that the composition of intestinal flora in DC patients was significantly different from that of negative controls; *Clostridium* could reduce the abundance of intestinal flora and have a greater correlation with the pathogenesis of inflammatory bowel disease. DC patients with symptoms had a higher proportion of Ruminococcus and Clostridium in gut microbiota. These results provide a basis for the clinical interventional treatment for enterostomy status-related DC in patients with permanent stoma.

There were still some problems to be improved in this study, including:(1) The sample size of this study was small, and the scope was limited to patients with rectal cancer who received terminal ileostomy;(2) we have found that specific flora is closely related to DC, but animal experiments are needed in the later stage to clarify the causal relationship and mechanism;(3) The DC grade used in this study was based on the colonscopic scores, and more precise grade needs to be combined with the pathological results of the intestinal inflammatory tissue;(4) In addition to bacterium, there are fungi and viruses in the gut, and we need to follow-up through metagenomic sequencing to clarify the mechanism.

## 7 Conclusion

In a stoma state, DC patients show a few symptoms of the digestive tract but rather obvious colonscopic characteristics. Subsequent to colostomy closure surgeries, a series of serious clinical signs may appear. In addition to age, BMI, and diabetes influencing DC severity, colonscopic scores are also an independent risk factor related to diarrhea severity after the colostomy closure surgery. Moreover, DC patients with colonscopic scores at diverse grades are significantly different from each other in their local and systemic inflammatory responses, intestinal flora compositions, and diversity structures. In particular, obvious differences are found in the abundance of Bifidobacteriales, Prevotella, Providencia, and Dorea.

## Data availability statement

The data presented in the study are deposited in the GEO repository, accession number GSE226706.

## Ethics statement

The studies involving human participants were reviewed and approved by ChiECRCT-20180225. The patients/participants provided their written informed consent to participate in this study. Written informed consent was obtained from the individual(s) for the publication of any potentially identifiable images or data included in this article.

## Author contributions

QS wrote the manuscript. YS and XiaL collected specimens. HL, LZ, WW and WZ sorted out relevant materials and literature. YH provided theoretical guidance. ZH and XinLi reviewed the paper. All authors contributed to the article and approved the submitted version.
