# Supplementary material for: Interaction between the intestinal flora and the severity of diversion colitis after low anterior resection of rectal cancer
Source: Front Oncol. 2023 Mar 14;13:1001819. doi: 10.3389/fonc.2023.1001819 (PMC10043175; doi:10.3389/fonc.2023.1001819)
Supplement: Supplementary file 1 [file Table_1.docx]

**Table S1. Detailed grading rules for for colonscopic severity score of DC**

| Performance | Scoring rules | Total score |
| --- | --- | --- |
| Edema | None (0), Mild (1), Moderate (2), Severe (3) | 0~7 |
| Mucosal Hemorrhage | None (0), Mild (1), Moderate (2), Severe (3) |  |
| Contact Hemorrhage | None (0), Yes (1) |  |

**Table S2. 20-point evaluation method of diarrhea severity**

| Clinical performance | Score | Clinical performance | Score |
| --- | --- | --- | --- |
| Diarrhea duration（d） | | Body temperature（℃） | |
| 1~4 | 1 | 37.5~38.5 | 1 |
| 5 | 2 | 38.6~38.9 | 2 |
| ≥6 | 3 | ≥39.0 | 3 |
| Highest frequency of diarrhea  in a single day | | Dehydration state（%） | |
| 1~3 | 1 | 1~2 | 1 |
| 4~5 | 2 | 3~5 | 2 |
| ≥6 | 3 | ≥6 | 3 |
| Vomiting duration（d） | | Treatment measures |  |
| 1 | 1 | Outpatient treatment | 1 |
| 2 | 2 | Hospitalization | 2 |
| ≥3 | 3 |  |  |
| Highest frequency of vomiting  in a single day | |  |  |
| 1 | 1 |  |  |
| 2~4 | 2 |  |  |
| ≥5 | 3 |  |  |

**Table S3. Table of OTU cluster draw**

| Sample name | Tag number | OTU number | Sample name | Tag number | OTU number |
| --- | --- | --- | --- | --- | --- |
| 1 | 50963 | 185 | 21 | 54524 | 100 |
| 2 | 50879 | 162 | 22 | 58710 | 87 |
| 3 | 52911 | 106 | 23 | 52355 | 126 |
| 4 | 56967 | 98 | 24 | 54597 | 159 |
| 5 | 54403 | 110 | 25 | 56108 | 73 |
| 6 | 53441 | 66 | 26 | 58431 | 49 |
| 7 | 53106 | 172 | 27 | 58111 | 165 |
| 8 | 54485 | 71 | 28 | 53238 | 202 |
| 9 | 57017 | 222 | 29 | 59777 | 114 |
| 10 | 53078 | 99 | 30 | 54719 | 122 |
| 11 | 58927 | 64 | 31 | 54862 | 97 |
| 12 | 58204 | 187 | 32 | 55318 | 105 |
| 13 | 56267 | 129 | 33 | 56698 | 170 |
| 14 | 58053 | 120 | 34 | 59376 | 144 |
| 15 | 58180 | 159 | 35 | 58952 | 84 |
| 16 | 57741 | 127 | 36 | 55222 | 98 |
| 17 | 49047 | 118 | 37 | 57186 | 155 |
| 18 | 55615 | 131 | 38 | 54585 | 133 |
| 19 | 56232 | 98 | 39 | 57666 | 125 |
| 20 | 54784 | 136 | 40 | 60074 | 73 |
