# Supplementary material for: Interaction between the intestinal flora and the severity of diversion colitis after low anterior resection of rectal cancer
Source: Front Oncol. 2023 Mar 14;13:1001819. doi: 10.3389/fonc.2023.1001819 (PMC10043175; doi:10.3389/fonc.2023.1001819)

Figure S1. Examples of endoscopic scoring of different severity in DC

Note: A-C: Mucosal Hemorrhage (A: 1; B: 2; C: 3) ; D-F: Edema (D: 1; E: 2; F: 3) ; G-I: Contact Hemorrhage

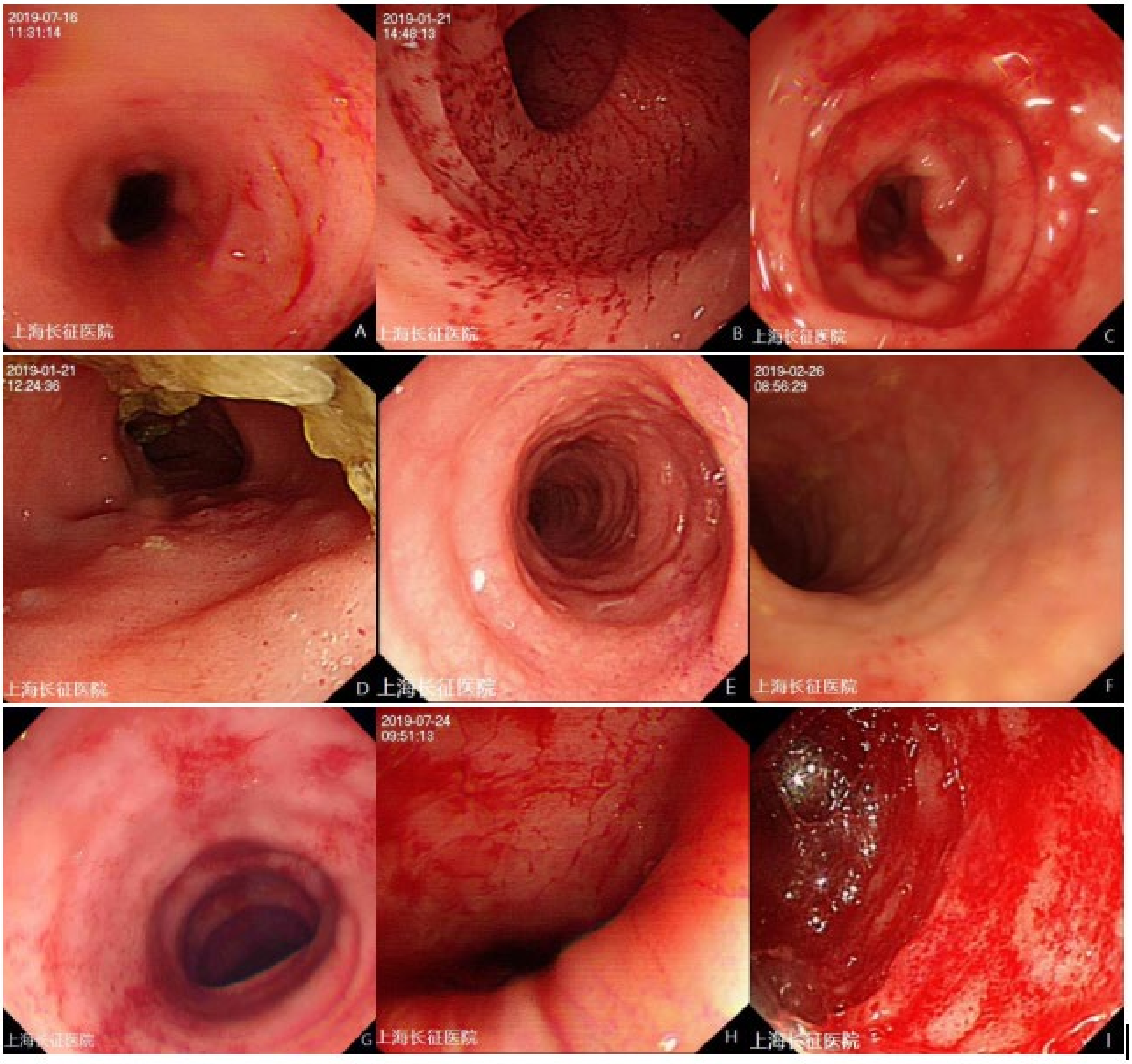

Supplement: Supplementary file 2 [file Image_1.pdf]
